# Older Adults’ Perceptions and Attitudes Toward Passive Sensors to Measure Loneliness: A Qualitative Study

**DOI:** 10.1177/30495334251325607

**Published:** 2025-04-29

**Authors:** Emma Cho, Hannah Cho, George Demiris, Sean Harrison, Xiayan Ji, Ahhyun Yuh, Oleg Sokolsky, Insup Lee

**Affiliations:** 1 The University of Pennsylvania School of Nursing, Philadelphia, USA; 2 University of Pennsylvania School of Engineering and Applied Sciences, Philadelphia, USA

**Keywords:** technology, loneliness, older adults

## Abstract

**Background:**

Loneliness among older adults significantly affects their health, increasing the risk of mortality, cardiovascular disease, and cognitive decline. Approximately 43% of American adults aged 60 and older report loneliness. Technology-based solutions such as passive sensors offer innovative ways to monitor and alleviate loneliness. In addition to understanding older adults’ overall attitudes toward passive sensing, it is important to examine how they may perceive such technologies specifically for the assessment of loneliness. This study examines older adults’ perceptions and attitudes toward the use of passive sensors to measure loneliness, with the goal of understanding the feasibility and acceptability of the technology.

**Methods:**

A single-group, longitudinal, observational study was conducted with 17 older adults aged 65 and older living independently in Philadelphia. Seven types of sensors were installed in participants’ homes for 6 months to monitor motion, proximity, temperature, television viewing, sleep quality, and physical activity. Participants completed the UCLA Loneliness Scale and were interviewed after the study about their experiences with the sensors. Thematic analysis was used to identify key attitudes and perceptions, and SPSS was used for demographic analysis.

**Results:**

The mean age of the participants was 73.5 years (range 67–84). Key themes that emerged from the analysis include participants’ adaptation and adjustment, privacy and trust in data sharing, and perceived benefits and concerns of the installed sensors. Benefits included detecting and reducing loneliness, monitoring physiological parameters, improving healthier behaviors, etc. Concerns about privacy were raised, such as potential misuse by authorities or third parties, lack of accuracy of functions or feasibility of sensors, and lack of human response.

**Discussion:**

Passive sensors were generally accepted, but raised concerns about data security and skepticism about measuring emotional states. Transparent communication and education are needed to address these issues. Improved sensor designs that are more user-centered could encourage adoption, and the technology shows promise for both measuring and potentially alleviating loneliness (e.g., through prompting increased activity).

## Translational Significance

To address the critical challenge of loneliness among older adults, which significantly increases their risk of mortality and chronic disease, this study explores the use of passive sensors to monitor indicators of loneliness. Findings reveal a general acceptance of this technology among seniors, along with concerns about privacy and data security. By improving sensor design and ensuring transparent communication, these devices could provide a viable solution for detecting and mitigating loneliness, thereby improving health outcomes and quality of life for the elderly population.

The prevalence of loneliness and social isolation among older adults is a serious problem in today’s society. Approximately 24% of community-dwelling Americans aged 65 and older experience social isolation (Cudjoe et al., 2020), and 43% of older adults aged 60 and older report experiencing loneliness (National Academies of Sciences, Engineering, and Medicine, 2020). The effects of this phenomenon go beyond mere emotional discomfort and pose a serious threat to the physical health and well-being of older adults. Research continues to demonstrate the link between loneliness in older adults and a range of negative health outcomes, including increased mortality (Perissinotto et al., 2012), increased risk of cardiovascular disease, and accelerated cognitive decline (Cacioppo et al., 2014). Despite growing awareness of the impact of loneliness, addressing loneliness and social isolation among older adults remains a daunting challenge, exacerbated by the dynamics of modern society and the aging of the global population.

Social isolation and loneliness are often used interchangeably, but they are distinct concepts. Social isolation refers to the objective state of having few social relationships or infrequent social contact with others (Gardner et al., 1999), whereas loneliness refers to the subjective feeling of being isolated (Peplau & Perlman, 1981). Loneliness has been measured in research using self-report questionnaires that assess individuals’ subjective experiences of social connectedness and emotional well-being. Among the most widely used instruments is the UCLA Loneliness Scale, which includes a series of statements about feelings of loneliness and social isolation to which respondents indicate their level of agreement (Russell, 1996). Another commonly used measure is the De Jong Gierveld Loneliness Scale, which distinguishes between emotional and social loneliness, providing a more nuanced understanding of the loneliness experience (De Jong Gierveld & Van Tilburg, 2006).

These self-report measures provide valuable insights into individuals’ perceived levels of loneliness, however they are subject to potential biases that arise from the reliance on participants’ willingness and ability to accurately reflect and report their feelings, which can be influenced by social desirability, recall problems, or discomfort with disclosing negative emotions (Althubaiti, 2016; Paulhus, 1984; Polit & Beck, 2021). These factors can obscure a precise understanding of loneliness, especially in vulnerable populations like older adults.

In recent years, researchers have employed a variety of sensing technologies to assess loneliness and social isolation among older adults, particularly in European countries such as Sweden (Azimi et al., 2017), Norway (Søraa et al., 2021), and Denmark (Yamazaki et al., 2014). These technologies have been instrumental in addressing the issue of loneliness among seniors by providing continuous, unobtrusive monitoring of behaviors often associated with social isolation. For example, motion sensors are used to analyze movement patterns in the living environment to capture subtle changes in behavior, such as decreased mobility or irregular activity, which are often indicators of social withdrawal or deteriorating mental health (Ji et al., 2023). Devices such as fitbits and mattress sensors monitor sleep patterns and physical activity (Doryab et al., 2019), which can provide valuable insights into sleep disturbances and decreased social engagement, which are strongly associated with loneliness and social isolation (Chen et al., 2021; Luo et al., 2020). Similarly, wearables with pedometers and gyroscopes provide continuous data on physical activity levels, allowing researchers to track daily activity patterns to assess the extent of social isolation (Cooper et al., 2018).

A recent study by Ji et al. (2024), which evaluated 22 sensor features in senior communities in the United States and Japan, found that sleep mattress sensors and temperature and humidity sensors were the most effective at predicting loneliness. These findings suggest that non-invasive sensors can serve as efficient and reliable tools for measuring loneliness in older adults.

While the benefits of these technologies are increasingly recognized, it is important to understand older adults’ perceptions to ensure successful adoption and integration. In a study of perceptions of contactless monitoring, although not limited to loneliness-specific monitoring, older adults reported that contactless monitoring was useful for living safely and independently at home for longer and for timely detection of emergencies and gradually developing health problems. Acceptance of sensor technology was found to be influenced by its potential usefulness, the usefulness of the information collected, the functional requirements of the system, and its financial cost (Claes et al., 2015). However, significant gaps remain in our understanding of how older adults perceive technologies designed to monitor loneliness. Addressing these issues is essential for the development of ethically responsible and effective health technologies that respect older adults’ autonomy and preferences. Therefore, this study aims to explore older adults’ attitudes and perceptions toward the use of passive sensors to assess loneliness. The findings will help guide the development of tailored interventions that meet the needs and preferences of older adults, ultimately creating a more connected and supportive environment for the aging population.

## Methods

### Design

To assess older adults’ perceptions and attitudes toward the use of passive sensors to measure loneliness, we conducted a single-group longitudinal observational study. We recruited community-dwelling older adults who were willing to have passive sensors installed in their homes. The sensors were installed in the homes of 17 older adults from April to September 2023 and were designed to provide indirect indicators of loneliness by monitoring behavioral patterns associated with social isolation, such as decreased exercise, reduced social contact, and changes in daily routines. Participants were encouraged to complete the 20-item UCLA Loneliness Survey on a weekly basis and were informed that the sensors would not directly measure loneliness, but would track behaviors that could correlate with loneliness, such as time spent alone or changes in sleep and activity patterns. At the end of the study, participants were interviewed about their thoughts on the system and its usability, and their experience living with the sensors.

### Participants

Potential participants were recruited from the University of Pennsylvania School of Nursing area by contacting staff members of senior living communities. All potential participants interested in the study were screened for eligibility: they had to be at least 65 years old, have no significant cognitive impairment (as assessed by the Mini-Mental State Examination; Folstein et al., 1975), have access to the Internet/Wi-Fi, and live independently in the University of Pennsylvania School of Nursing area. Exclusion criteria were not meeting the above eligibility requirements and being unable to speak English. In total, 17 recruited participants provided informed consent after receiving a full explanation of the consent form. The study was approved by the University of Pennsylvania School of Nursing.

### Technology

Participants were asked to provide permission to the research team to install seven different types of sensors in their homes for 6 months. These sensor types reflected the nuances of mental health and socialization in older adults and included motion and contact sensors known to track movement patterns, BLE tag-based proximity sensors, temperature and humidity sensors, electricity meter sensors, sleep mattress sensors, and activity trackers.

Motion and contact sensors recorded daily movements or door openings in specific areas (entryway, kitchen, bathroom), BLE tag-based proximity sensors recorded whether participants were home or away, and temperature and humidity sensors extracted average temperature, average humidity, and number of shower events in participants’ homes. The power meter sensor was connected to the participant’s TV and measured the number of hours they watched TV during the day; the sleep mattress sensor measured sleep efficiency, sleep latency, total sleep time, and nap time; and the activity tracker measured average weekly steps.

### Measures

Participants completed basic demographic information (e.g., age, gender, education, and race/ethnicity) and the University of California Los Angeles (UCLA) Loneliness Scale Version 3, which served as the primary tool for directly assessing loneliness, while the sensors provided supplemental data on behavioral patterns that could be associated with loneliness. The UCLA Loneliness Scale is a 20-item instrument in which each item is scored from 1 to 4, with 1 = never, 2 = rarely, 3 = sometimes, and 4 = often. Positive statements (items 1, 5, 6, 9, 10, 15, 16, 19, and 20) were reversed and scored. Possible total scores for this scale ranged from 20 to 80, with higher scores indicating greater levels of loneliness (Russell, 1996). Participants completed the UCLA Loneliness Scale weekly.

At the end of the study, a trained and experienced researcher conducted semi-structured, individual interviews with participants at exit, following a standardized interview guide (see Appendix 1). All interviews (30 min to 1 hr) were audio-recorded and transcribed. The interviewer guide included questions about the participants’ overall experience with the sensors, their perceptions of how well the sensors integrated into their daily lives, any challenges or discomfort they encountered, their understanding of the sensor’s purpose, and their views on the sensors’ effectiveness in reflecting their feelings of loneliness.

### Data Analysis

Basic descriptive data from the participants were analyzed using SPSS. Descriptive statistics were used to analyze participants’ average loneliness scores and characteristics, and correlation analysis was used to analyze the correlation between the sensor data and loneliness. Thematic analysis, which is a useful method for examining the perspective of different research participants, and generating unanticipated insights (Nowell et al., 2017), was used to explore the attitudes and perceptions of passive technology to measure loneliness in older adults.

The three authors (Cho, Emma; Cho, Hannah; Demiris, George) first familiarized themselves with the data by listening to the interview audio and reading the transcriptions. They then discussed and finalized the coding of themes related to users’ experiences with passive technology. The first author (Cho, Hannah) read the baseline interview transcriptions and wrote initial codes. After the three authors (Cho, Emma; Cho, Hannah; Demiris, George) reviewed the initial codes and reached consensus, the first author (Cho, Emma) developed a codebook that was used to systematically code all transcripts.

All codes and quotations were tabulated; each code and quotation were reviewed and discussed by the three authors to ensure accuracy, and themes were selected to capture sufficient details of thick descriptions of participants’ perceptions and attitudes toward the use of passive sensors to measure loneliness.

## Results

A total of 17 older adults over the age of 65 participated. The mean age of the participants was 73.5 years (*SD* = 5.1, range = 67–84). Of the participants, 76.5% were female, and 52.9% were African American (See Table 1). In terms of educational background, 64.6% had some college education, 17.6% held a bachelor’s degree, 11.8% had completed graduate-level education, and 5.9% had a high school diploma as their highest level of education (See Table 1). The average loneliness score among participants was 43.6, indicating a moderate level of loneliness within the participants.

**Table 1.** Demographic of the Study Participants.

Demographic factors	*n* (%)	Mean (*SD*)
Age		73.5 (5.1)
Gender		
Male	4 (23.5)	
Female	13 (76.5)	
Education		
High school	1 (5.9)	
Some college	11 (64.7)	
Bachelors degrees	3 (17.6)	
Graduate degrees	2 (11.8)	
Race/ethnicity		
White	7 (41.2)	
African American	9 (52.9)	
Other	1 (5.9)	

The thematic analysis revealed the following categories in which passive sensors for measuring loneliness were perceived as convenient and useful:

### Normalization

Most of participants reported that the sensors fit seamlessly into their home environment and were almost unnoticeable in their daily lives. For example, one participant highlighted the unobtrusive nature of the technology, stating, “I had absolutely no problem with it at all. It was non-invasive. . .Painless” suggesting a smooth transition to living with the sensors.

Despite the overall ease of integration noted above, some people experienced initial difficulties with the visual cues of the sensors, especially when moving around at night: “At first, I thought I had to get used to the flash going off at night, and then I realized it was kind of annoying, so it took me a while to mentally adjust to it” and “Every time I would come out here in the middle of the night, those things flashing in my eyes, I didn’t like that on the doorway and everything.” which sums up the discomfort some participants experienced until they became accustomed to the presence of the sensors.

### Self-Awareness and Behavior Change

The sensors acted as a motivator for increased physical activity and self-awareness among participants. One noted, “The sensors helped me maximize my activity. I walked six or seven miles at a time” demonstrating the potential of the technology to encourage healthier lifestyles. This was reinforced by another participant who appreciated the goal-setting aspect: “I started walking more because of it. . .And that was good” demonstrating the role of sensors in promoting positive behavior change.

### Confidence in Data Sharing

In terms of using the data collected from the sensors, a subset of participants expressed a high level of trust in healthcare professionals accessing their data, seeing it as an opportunity for improved health monitoring and intervention. “I have a therapist that I see. I would give her the information” and “That would be okay. Because then they can help me identify the times when I feel lonely. . .” suggest a willingness to share data in the interest of personal health and well-being.

Another participant voiced a broad sense of confidence about the transparency of their data, applicable to a wide array of potential viewers. “I don’t have anything that I need to fear somebody knowing. I’m just a normal person trying to make it day to day,” indicating a general comfort with sharing personal information.

However, attitudes toward data sharing were not uniformly permissive across all interpersonal relationships. Participant 27 expressed a more guarded stance when considering who should have access to their information. “I might be a little selective about friends, family, and whatever, you know,” they noted, highlighting a distinction in trust levels.

### Benefits and Utility

Participants generally acknowledged the value of the sensors for monitoring health metrics and encouraging physical activity. For example, one individual noted the sensors’ role in self-improvement, stating, “It helps a lot, it tells you what you need to improve on.” This reflects a recognition of the technology’s potential to provide actionable insights into one’s health and lifestyle.

Conversely in the thematic analysis, participants expressed inconvenience, concerns and expectations regarding the use of passive sensors to measure loneliness as summarized by the following themes:

### Concerns About Privacy and Surveillance

Some participants also had concerns about the clarity and intent of the data collection, as well as fears of potential misuse. Comments such as, “My concern is what kind of information are they collecting and what are they collecting it for?” and criticisms of surveillance culture, such as “I’ve heard some people say it’s like Big Brother watching. . .” reflect anxieties about privacy issues.

### Skepticism

Anxiety about misuse also led to skepticism about the ability to accurately measure complex emotional states such as loneliness; for example, comments such as “the system can’t detect it, it can’t think about it, it can’t feel it, the whole thing is a waste of money” illustrate doubts about the validity and effectiveness of using technology to measure emotional well-being.

### Expectation of Technology

While older adults’ perceptions of using the sensors were divided, their expectations for how the sensors should work also varied, and they called for improvements to make the technology more interactive and user-friendly. For example, suggestions included having the system provide daily feedback and setting reminders to keep participants engaged with the sensors. This feedback demonstrates a desire for a more personalized and responsive technology experience.

Overall, while the sensor technologies were largely accepted and even appreciated for their potential to encourage healthier behaviors and provide valuable data related to loneliness, concerns about privacy and data security remained prominent.

## Discussion

To our knowledge, this is the first study to examine older adults’ perceptions and attitudes toward passive sensors used to specifically measure loneliness. This study is unique in that it adds to the literature on older adults’ perceptions and attitudes toward technology that has previously been used to alleviate loneliness. In addition, the findings may provide new insights into the prevention of loneliness in older adults through the use of sensors. Participants’ reactions to the sensors varied, with some reporting high levels of acceptance because the sensors were barely noticeable, while others found them intrusive because of the light they emitted at night. These differences highlight the personal nature of technology adoption and suggest that minimizing the intrusiveness of sensors, particularly with respect to light emission, should be a design priority to increase acceptance among older adults.

Interestingly, in addition to function of sensor awareness, older adults also reported behavioral changes as a result of being aware of the sensor technology. In particular, the sensor that measured physical activity motivated participants to increase their activity and increased their self-awareness. While the primary purpose of the sensor was to measure loneliness, the secondary effect of encouraging increased physical activity may itself alleviate loneliness, which is supported by previous research showing that increased physical activity reduces loneliness in older adults (Musich et al., 2022). This dual role of sensors in measuring and alleviating loneliness highlights the multiple benefits of integrating these technologies into the lives of older adults.

Despite recognizing the potential health benefits of sensor technology, some older adults expressed concerns about privacy and clarity of data use intentions, which aligns with previous literature on older adults’ attitudes toward technology used to actively reduce loneliness (Chi et al., 2017; Hudson et al., 2020; Ten Bruggencate et al., 2019; Van Assche et al., 2023). For example, in a study of digital adoption among older adults, Chi et al. (2017) found that older adults had similar concerns about data security and the potential misuse of personal information in digital pet avatars used to augment their social interactions. These findings are consistent with those of Hudson et al. (2020), who reported that older adults are often uncomfortable with the opaque nature of data processing in technological interventions. However, as highlighted by Van Assche et al. (2023), there is also a contrasting view that some older adults are willing to adopt these technologies as long as transparent and direct communication about data processing and monitoring is maintained.

These mixed responses underscore the importance for transparent communication about data processing and surveillance purposes to facilitate technology acceptance. Furthermore, skepticism was also expressed about the ability of passive sensors to accurately measure complex emotional states such as loneliness, suggesting the need to address gaps in understanding by providing thorough information sessions about technology use. Thus, further studies should investigate how emotional monitoring could be implemented in a less invasive manner that provides users with clear feedback on their emotional states without causing discomfort.

Conversely, some older adults were open to sharing loneliness data with healthcare providers, believing that such information could enable timely and appropriate interventions. This perspective demonstrates that older adults recognize the potential for technology to facilitate better health outcomes, provided that concerns about privacy and data use are properly addressed.

Participants also offered useful suggestions for sensors, including the ability to encourage ongoing physical activity and provide feedback on behaviors that may exacerbate loneliness. These findings show that many older adults recognize the importance of using technology to combat loneliness and are taking a proactive approach to their own health and well-being.

While this study provides valuable insights into perceptions and attitudes of older adults toward monitoring technologies, it is important to acknowledge certain limitations that might affect the generalizability of the findings. First, the sample for this study was recruited from a population of retired educated older adults and mostly among White and African Americans. Therefore, the findings may not be generalizable to other groups of older adults. Second, the interviews were conducted only once at the study’s conclusion and were relatively brief. As such, the data may not fully capture the breadth of participants’ experiences over the 6-month period. Future research would benefit from more frequent interviews or longitudinal qualitative assessment to gain a deeper understanding of how participants’ perceptions evolve over time. Third, this study is limited in that it doesn’t delve deeply into how older adults feel about being monitored for their emotional states compared to their physical activity. This lack of detailed feedback makes it difficult to draw strong conclusions about their emotional responses to such monitoring. Future research should address this gap by including more direct questions about emotional monitoring in the study design. Additionally, while there continues to be a growing desire and effort to integrate technology into the daily lives of older adults, the lack of ethical guidelines to support technology development can sometimes treat users as dependent individuals rather than enhancing their autonomy, social participation, and independence.

Despite these limitations, the study provides valuable insights. It highlights how sensor technology can be used not only to track loneliness, but also to promote physical activity, which can help reduce feelings of loneliness. Therefore, new technology developments need to be better tailored to the needs of older adults by identifying their impact on individuals, and it is important to reduce older adults’ concerns about privacy by creating clear ethical guidelines.

## Conclusion

This study provides insight into older adults’ perceptions and attitudes toward passive sensor technology and, despite the limited sample size, can inform technology designers and healthcare providers. Going forward, it is necessary for system designers and new technology developers to iteratively assess and refine the needs and expectations of older adults to achieve a user-centered design that will increase usability and adoption.

## Appendix 1: Semi-Structured Interview Guide

Participant ID: Data:

### Introduction

Thank you for agreeing to participate in this interview. The purpose of this session is to gain insight into your perceptions and experiences regarding the use of sensor technology to measure loneliness and improve our intervention strategies. Please note that there are no “right” or “wrong” answers; we value your unique perspective and experiences.

The interview should take between 30 min and an hour, depending on the depth of information you are willing to share. With your permission, I would like to audio record the session to ensure that I accurately capture all of your comments. All answers will be kept confidential. This means that all personal information will be stored securely and only de-identified data will be shared with members of the research team. The final report will not contain any identifiable information that can be linked to you.

Before we continue, I would like to give you a brief refresher on the sensor technologies included in this study: the motion sensor, the contact sensor on the refrigerator and front door, the scale, the sleep mat, the tile tag, the temperature sensor, the fitness tracker, and the TV plug. You have the full right to skip any question, and you may stop the interview at any time if you so choose. If you have any questions or need further clarification on any aspect mentioned, please feel free to ask me.

*Please note that this guide only outlines the main topics for discussion with participants and does not cover the various prompts that might be used, although examples are provided for each question. In addition, non-leading and broad prompts such as “Can you tell me a little more about this?” and “What does this look like to you?” are used to facilitate discussion.*

### Establishing Rapport

Before we dive into the specifics, I’d like to hear about your general experience with the sensor technologies in your home. (Tailor a question here to a specific person and/or situation. e.g: “Could you share your thoughts on how you’ve found using these devices? What has been your overall experience with the sensors installed in your home?”)

1. General opinion about devices
How do you feel about having all these devices in your apartment/house?
**Prompts:** How do you feel about the number and type of devices installed? Has their presence affected your living experience?
2. Concerns about data and device operation
Do you have any concerns about how the data is collected or how the devices operate? If so, please elaborate.
**Prompts:** Are there specific privacy or security concerns?
3. Technology in Daily Life
What do you think about using technology to measure activities of daily living?
**Prompts:** How do you perceive the benefits versus intrusions of such measurements? Can you foresee long-term benefits of using this technology?
4. Changes in daily routine
What about your daily routine have you changed or adjusted because of the devices?
**Prompts:** Were these changes positive or negative? How easy or difficult was it to adjust to these changes?
5. Challenges and problems
What challenges or problems did you experience?
**Prompts:** Were there any technical difficulties with the equipment? How did you overcome these challenges?
6. Impact on home environment and personal space
How do you feel the devices/sensors may have interfered with your home environment or personal space?
**Prompts:** Did the devices interfere with the aesthetics or comfort of your home? How did you feel about the visibility of these devices to others?
7. Social interactions
How did the device affect your interactions with others?
**Prompts:** Did visitors notice or ask you about the devices? How did you explain the purpose of the devices to others?
8. Independence and agency
Did the sensor affect your sense of independence?
**Prompts:** Please elaborate on any feelings of dependence or empowerment due to the devices.
9. Alert system
How would you feel if you received alerts or notifications from a sensor system that indicated you might be feeling lonely?
**Prompts:** Would you be concerned if these alerts were not accurate? How would you prefer to be alerted without causing distress?
10. Sharing information with others
Would you be comfortable sharing information from such a loneliness monitoring system with your doctor or others who need to know?
**Prompts:** How could this information be helpful? Are there any privacy concerns you would like addressed?
11. Additional feedback
Do you have any other comments or feedback you’d like to share about the study or your experience?
**Prompts:** What aspects of the study did you find most useful or challenging? Are there any additional supports or improvements you would suggest for future studies?
